# POEMS Syndrome Presentation with an Abscess within the Plasmacytoma—A Rare Case Report

**DOI:** 10.5402/2011/173164

**Published:** 2011-03-30

**Authors:** Rishi Agarwal, Muneer H. Abidi, Bala Grandhi

**Affiliations:** ^1^Department of Internal Medicine, Synergy Medical Education Alliance, Michigan State University College of Human Medicine, Saginaw, MI 48602, USA; ^2^Division of Hematology and Oncology, Wayne State University and Karmanos Cancer Institute, Detroit, MI 48201, USA

## Abstract

POEMS Syndrome is a rare cause of demyelinating and axonal mixed neuropathy. Plasmacytomas are usually seen in POEMS syndrome and can be osseous or extramedullary. Plasmacytomas presenting as an abscess has not been noted earlier. Our patient presented with localized hyperpigmented patch on the back and later developed progressive weakness in upper and lower limbs. Initially serum and urine protein electrophoresis were normal. The patient was thought to have Chronic Inflammatory Demyelinating Polyneuropathy and was treated accordingly without any improvement. Repeat serum protein electrophoresis showed monoclonal gammopathy. MRI of the back revealed an abscess in the paravertebral soft tissues reaching up to the skin. Needle biopsy was consistent with plasmacytoma. Later, he developed a purulent fungating lesion in the lower midback. Antibiotics were started and local resection was done followed by radiation. Pathology of the resected mass showed plasmacytoma extensively involving subcutaneous soft tissue and bone. The patient improved with the treatment. Cystic plasmacytomas and abscess within the plasmacytoma has not been reported earlier. Whether abscess formation is part of the disease spectrum due to infiltration of overlying tissue or is secondary to localized immunosuppression is unknown. Local treatment of a single plasmacytoma is useful in ameliorating systemic symptoms.

## 1. Background

POEMS syndrome also known as osteosclerotic myeloma, Crow-Fukase syndrome [1] or Takatsuki and Sanada [2] syndrome is a rare cause of demyelinating and axonal mixed neuropathy with multiorgan involvement and monoclonal plasma cell proliferative disorder [3]. It was first described in 1968 by Shimpo [4] and was later given its name by Bardwick et al. [5]. The acronym POEMS is indicative of Polyneuropathy, Organomegaly, Endocrinopathy, Monoclonal protein and Skin changes [5]. POEMS syndrome commonly presents chronic progressive polyneuropathy with a predominant motor disability [1–7]. Osteosclerotic bone lesions with the evidence of plasma cells infiltration (plasmacytomas) are commonly seen in POEMS syndrome [1–7]. Solitary osseous plasmacytoma without other features of POEMS syndrome usually presents as a lytic lesion. Extramedullary plasmacytomas are relatively rare [8, 9].

Plasmacytomas presenting as an abscess have not been noted earlier. This could be a manifestation of the localized immunosuppression. Immunosuppression in POEMS syndrome has not been well described in literature and bacterial infections as initial presentation has not been seen. 

We are presenting a unique case of POEMS syndrome with a solitary plasmacytoma which presented as an abscess involving the spinous process and overlying skin. The patient's symptoms improved significantly after the resection of plasmacytoma.

## 2. Case Presentation

A 51-year-old African American obese male with history of diabetes and hypertension presented with a localized hyperpigmented patch on the back which was biopsied and was found to be interstitial dermatitis with inflammatory morphea. It was thought to be a benign lesion, and the patient was started on a topical steroid cream. No other intervention was done at that time. About nine months later, he came back with the complain of progressive weakness in upper and lower limbs and frequent falls. He was unable to walk without a walker. He also had tingling, numbness, and burning in his feet. It was also noted at that time that his skin patch in the back has increased in size and he had another hyperpigmented patch in right-lower quadrant of his abdomen.

On physical examination he had stable vital signs. He had a hyperpigmented patch with regular borders approximately 9 × 2.5 cm on the right lower quadrant of the abdomen and a hyperpigmented patch with hyperkeratosis on the lower midback measuring about 47 × 15 cm. He also had hepatomegaly. On neurological exam he had motor and sensory loss in the extremities. His power was 4/5 at elbow bilaterally on both flexion and extension and 4/5 at the knee bilaterally on flexion and extension.

The patient's initial workup showed platelet count of 633 k/*μ*L (normal 130–400 k/*μ*L). Lumbar puncture was grossly acellular with abnormal glucose (139 mg/dL, normal 40–70 mg/dL) and protein (99 mg/dL, normal 10–60 mg/dL). Initially serum and urine protein electrophoresis as normal. Electromyogram was done, which showed demyelinating neuropathy with evidence of conduction block and temporal dispersion. Bone marrow biopsy did not show any atypical plasma cells. Plasmacytosis (5%) was seen on bone marrow examination. 

The patient was initially thought to have chronic inflammatory demyelinating polyneuropathy (CIDP) and was started on high-dose steroids, and later intravenous immunoglobulin (IVIG) was given. There was no improvement in his condition. 

**Figure 1:**
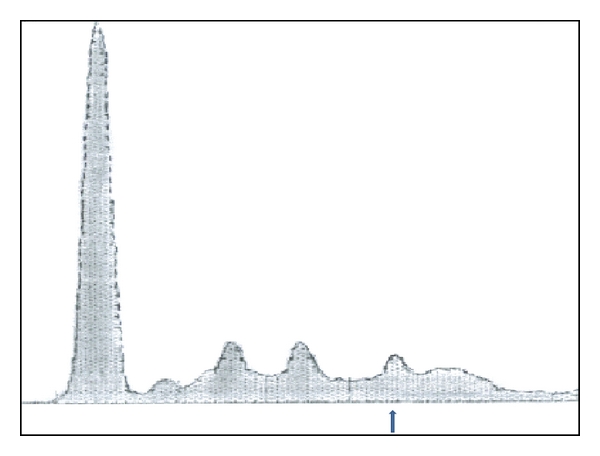
Serum protein electrophoresis of the patient showing IgG spike (arrow).

Repeat serum protein electrophoresis showed small gamma spike and monoclonal gammopathy (IgG type lambda 19.4% (normal 9%–18%)).Urine electrophoresis showed light chain M protein (Figure 1). 

**Figure 2:**
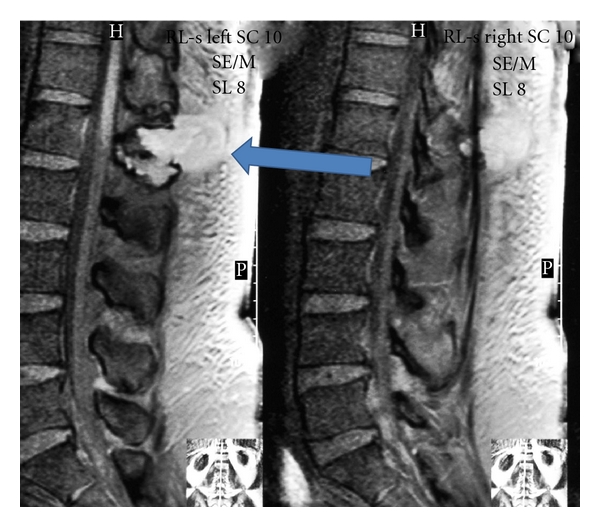
MRI of lumbar spine showing abscess at L1 (arrow).

MRI of the back was done to look for spinal causes of leg weakness which incidentally revealed 6 cm × 3.5 cm × 4 cm abscess in the posterior paravertebral soft tissues reaching up in the skin causing inflammation around it with osteomyelitis of the L1 spinous process. No spinal nerve compression or epidural involvement was noted (Figure 2). 

Fluoroscopy-guided aspiration of the abscess revealed hemorrhagic fluid which did not grow anything on culture. patient reported no change in his condition. The patient was still being treated as CIDP, and he was started on plasmapheresis for 10 days. At this point, it was thought that the gammopathy was probably monoclonal gammopathy of undetermined significance.

The patient continued to worsen neurologically. A whole body X-ray scan was done which revealed a lytic lesion at L2. Subsequent to that a CT scan to evaluate the lytic lesion and CT-guided fine needle aspiration (FNA) of the mass growing from L1 spinous process was done which was consistent with plasmacytoma on pathology. After the FNA he developed an infected purulent discharging fungating lesion in the lower midback at the site of hyperpigmentation. He was started on piperacillin/tazobactam and vancomycin. Antibiotics were narrowed down to ciprofloxacin and later piperacillin based on the culture results which grew *Pseudomonas* and coagulase-negative *Staphylococcus*. A repeat MRI of the back showed interval increase in size of the abscess noted previously. 

**Figure 3:**
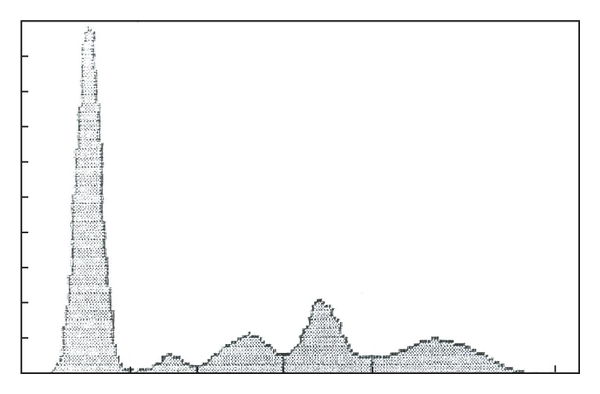
Recent serum protein electrophoresis of the patient showing resolution of IgG spike.

The patient underwent lumbar subcutaneous mass excision, L1 spinous process excision, and partial laminectomy. Pathology of the resected mass from L1 spinous process showed plasmacytoma extensively involving subcutaneous soft tissue and portions of bone. He also received radiation to the lumbar area. He was continued on piperacillin for four more weeks after discharge. The patient responded well to the treatment and showed increased strength in extremities. Skin changes on his back and abdomen also resolved. Thrombocytosis resolved and gamma proteins decreased from 1.4 g/dL to 0.9 g/dl (Ref. range 0.5–1.4 g/dl). He later developed weakness in upper arms with discovery of a lytic lesion in the cervical spine for which he received 4000 cGy radiation using intensity-modulated radiation therapy. The patient has apparently been stable since his radiotherapy which was done around 8 years ago. His recent serum protein electrophoresis is normal (Figure 3).

## 3. Discussion and Conclusion

Plasma cell neoplasms account for approximately 1% to 2% of human malignancies and occur at a rate of about 3.5/100,000 per year [8]. Less than 10% of patients with plasma cell neoplasms present with a solitary plasmacytoma that may be either extramedullary or osseous [8, 9]. Extramedullary plasmacytoma (EMP) is a rare disease which typically presents as a well-localized submucosal mass or swelling. EMPs often have polypoidal configuration and are more common in head and neck region [9]. Cutaneous plasmacytomas are rare EMPs and can be primary or secondary. The secondary cutaneous plasmacytomas arise from underlying bony lesion. 

Plasmacytomas are known manifestations in POEMS syndrome and can be typically differentiated from solitary plasmacytomas by the presence of other clinical features and also by the presence of osteosclerotic bony lesions in POEMS syndrome instead of only osteolytic lesions in solitary plasmacytomas.

Our patient, who presented with neuropathy and skin changes, had an abscess at L1 spine which was detected incidentally on the MRI which was performed to look for spinal compression and to rule out other causes for lower extremities weakness. The biopsy of the lesion confirmed that it was a plasmacytoma. 

Cystic plasmacytomas and abscess arising within the plasmacytoma and underlying osteomyelitis as seen in our patient has not been reported earlier, and to the best of our knowledge, this is the first such case reported. Whether abscess formation is part of the disease spectrum due to infiltration of overlying tissue or is secondary to localized immunosuppression is unknown. 

POEMS syndrome remains a difficult and rare diagnosis. Neuropathy is present in almost all the cases of POEMS syndrome. First-line treatment of neuropathic pain in plasma cell dyscrasias is Ca channel alpha (2) delta ligands (i.e., gabapentin and pregabalin) and certain antidepressants (i.e., tricyclic antidepressants and dual reuptake inhibitors of both serotonin and norepinephrine) [10].

In the case of solitary plasmacytoma early diagnosis, proper treatment with radiation is necessary to prevent unnecessary complications. Surgical resection can be considered as a possible treatment in case there are infectious complications such as an abscess as observed in our patient. As seen in our case local treatment of a single plasmacytoma with surgical removal and radiation is useful in ameliorating the systemic symptoms [11, 12]. Six weeks of antibiotic therapy was useful to treat the abscess and osteomyelitis. 

POEMS syndrome has a chronic course, and survival is three times greater compared to multiple myeloma. Median survival of POEMS syndrome is around 165 months, regardless of how many syndrome features, bone lesions, or plasma cells are present at the time of diagnosis. Additional features of the syndrome often develop in future, but the complications of classic multiple myeloma rarely develops. Even patients with multiple bone lesions or those with more than 10% plasma cells do not progress to overt multiple myeloma. Cardiorespiratory failure and pneumonia are the common causes of death in POEMS syndrome [6].

## Abbreviations

EMP: Extramedullary plasmacytomas,
CIDP: Chronic Inflammatory demyelinating polyneuropathy,
MRI: Magnetic resonance imaging,
FNA: Fine needle aspiration.
